# Decreased Expression of the *ARID1A* Gene Is Associated with Poor Prognosis in Primary Gastric Cancer

**DOI:** 10.1371/journal.pone.0040364

**Published:** 2012-07-13

**Authors:** Dan-dan Wang, Yi-bing Chen, Ke Pan, Wei Wang, Shi-ping Chen, Ju-gao Chen, Jing-jing Zhao, Lin Lv, Qiu-zhong Pan, Yong-qiang Li, Qi-jing Wang, Li-xi Huang, Miao-la Ke, Jia He, Jian-chuan Xia

**Affiliations:** 1 State Key Laboratory of Oncology in Southern China and Department of Experimental Research, Sun Yat-sen University Cancer Center, Guangzhou, People’s Republic of China; 2 Department of Gastric and Pancreatic Surgery, Sun Yat-sen University Cancer Center, Guangzhou, People’s Republic of China; Duke-National University of Singapore Graduate Medical School, Singapore

## Abstract

**Background:**

The *ARID1A* gene encodes adenine-thymine (AT)-rich interactive domain-containing protein 1A, which participates in chromatin remodeling. *ARID1A* has been showed to function as a tumor suppressor in various cancer types. In the current study, we investigated the expression and prognosis value of *ARID1A* in primary gastric cancer. Meanwhile, the biological role of *ARID1A* was further investigated using cell model in vitro.

**Methodology/Principal Findings:**

To investigate the role of *ARID1A* gene in primary gastric cancer pathogenesis, real-time quantitative PCR and western blotting were used to examine the *ARID1A* expression in paired cancerous and noncancerous tissues. Results revealed decreased *ARID1A* mRNA (*P* = 0.0029) and protein (*P* = 0.0015) expression in most tumor-bearing tissues compared with the matched adjacent non-tumor tissues, and in gastric cancer cell lines. To further investigate the clinicopathological and prognostic roles of *ARID1A* expression, we performed immunohistochemical analyses of the 224 paraffin-embedded gastric cancer tissue blocks. Data revealed that the loss of *ARID1A* expression was significantly correlated with T stage (*P* = 0.001) and grade (*P* = 0.006). Consistent with these results, we found that loss of *ARID1A* expression was significantly correlated with poor survival in gastric cancer patients (*P* = 0.003). Cox regression analyses showed that *ARID1A* expression was an independent predictor of overall survival (*P* = 0.029). Furthermore, the functions of *ARID1A* in the proliferation and colony formation of gastric cell lines were analyzed by transfecting cells with full-length *ARID1A* expression vector or siRNA targeting *ARID1A*. Restoring *ARID1A* expression in gastric cancer cells significantly inhibited cell proliferation and colony formation. Silencing *ARID1A* expression in gastric epithelial cell line significantly enhanced cell growth rate.

**Conclusions/Significance:**

Our data suggest that *ARID1A* may play an important role in gastric cancer and may serve as a valuable prognostic marker and potential target for gene therapy in the treatment of gastric cancer.

## Introduction

Gastric cancer is the fourth most common malignancy worldwide and the second most common cause of cancer-related deaths each year (10.4% of cancer deaths) [1]. Treatment of gastric cancer includes a combination of surgery, chemotherapy, or radiation therapy. However, nearly 60% of the patients affected succumb to gastric cancer even after a curative resection alone or after adjuvant therapy [2]. It has long been known that gastric cancer results from a combination of environmental factors and the accumulation of generalized and specific genetic alterations. Many of the genetic or epigenetic alterations associated with gastric cancer, including loss of heterozygosity, microsatellite and chromosomal instability and hypermethylation, have been reported [3]. Understanding these alterations and the molecular mechanisms involved in gastric carcinogenesis will be critical for the improvement of diagnosis, therapy and prognosis prediction of this disease.

The eukaryotically conserved SWI/SNF chromatin-remodeling complex plays essential roles in a variety of cellular processes, including differentiation, proliferation and DNA repair [4]. Loss of SWI/SNF subunits has been reported in most tumors, and a large number of experimental observations suggest that this complex is critical for tumor suppression [5]. The complexes contain seven or more noncatalytic subunits that presumably help modulate the targeting and activity of the ATPase [6]. One subunit of this complex, hSNF5/Ini1/BAF47, has been identified as a tumor suppressor [7], [8]. The other noncatalytic subunit, p270/ARID1A/BAF250a (adenine-thymine AT-rich interactive domain-containing protein 1A), has been demonstrated to be essential for normal cell cycle arrest [9]. Knockdown of *ARID1A* in a leukemia cell line confers resistance to Fas-mediated apoptosis [10].

Recently, *ARID1A* mutations and loss of BAF250a protein have been found to correlate strongly with the ovarian clear-cell carcinoma and uterine low-grade endometrioid carcinoma [11]–[13]. These observations indicate that *ARID1A* is a potential candidate tumor suppressor gene. However, the clinical significance of such differential expression and the function of the ARID1A protein remain undefined due to the lack of studies using fresh human tumor samples. In the present study, we analyzed the *ARID1A* expression level in gastric cancer using real-time quantitative RT-PCR, western blotting and immunohistochemistry. Meanwhile, we identified the relationship between *ARID1A* expression and clinicopathological features and evaluated its prognostic value in post-resection survival of gastric cancer patients. Furthermore, we evaluated the functional role of *ARID1A* in the tumorigenesis of primary gastric cancer by examining the in vitro proliferation and colony formation in gastric cell lines.

## Results

### *ARID1A* mRNA Expression Analyzed by Real-time Quantitative RT-PCR

**Figure 1:**
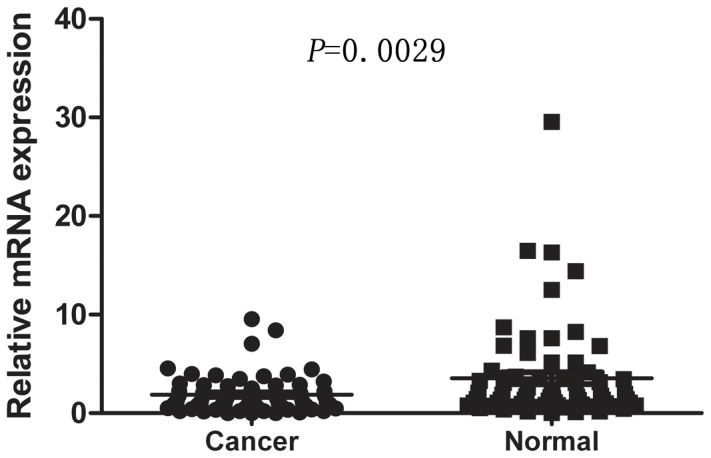
The mRNA expression of *ARID1A* in human primary gastric cancer surgical specimens was evaluated by real-time quantitative PCR. The relative mRNA expression of *ARID1A* was significantly decreased in gastric cancer tissues compared with the matched adjacent nontumorous tissues (n = 66, *P* = 0.0029). Horizontal lines represent the mean.

The mRNA level of *ARID1A* was determined by real-time quantitative RT-PCR assays in 66 paired cancerous and the matched adjacent normal gastric mucosa tissues. The *ARID1A* expression level was significantly lower in 43 (65.15%) tumor-bearing tissues compared with the adjacent non-tumor tissues (*P* = 0.0029, Figure 1).

### ARID1A Protein Expression Analyzed by Western Blotting

**Figure 2:**
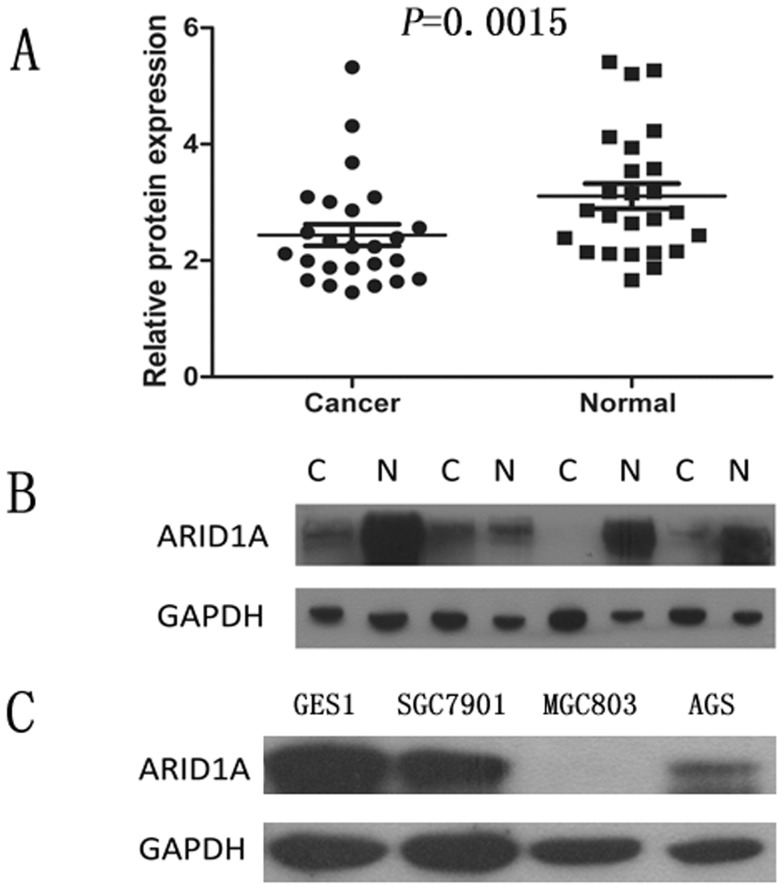
Decreased protein expression of ARID1A in gastric cancer as assessed by Western blotting. (A) Relative ARID1A protein expression levels in gastric cancer tissues and noncancerous tissues (ARID1A/GAPDH, n = 25, *P* = 0.0015). Horizontal lines represent the mean. (B) Representative result of ARID1A protein expression in 4 paired gastric tumorous and the matched adjacent nontumorous tissues (C, gastric cancer tissues; N, matched noncancerous gastric mucosa). (C) The ARID1A protein level was remarkably decreased in gastric cancer cell lines, SGC7901, AGS, especially in MGC803, compared with normal gastric cell line GES1.

Western blotting was performed on 25 gastric cancer specimens and corresponding adjacent non-cancerous gastric mucosa tissues from the 66 paired samples. The results showed an ARID1A band at the expected size of 242 kDa and the amount of ARID1A protein present was further measured by densitometry. Consistent with the quantitative real-time PCR results, a decrease in ARID1A expression was seen in 13 (52%) of the gastric tumor tissues compared with matched adjacent non-tumor tissues (*P* = 0.0015, Figure 2A and Figure 2B). Likewise, the ARID1A protein expression was remarkably decreased in gastric cancer cell lines, SGC7901, AGS, especially in MGC803, compared with normal gastric cell line GES1 (Figure 2C).

### Immunohistochemical Analysis of ARID1A Expression in Gastric Cancer Tissue Samples and its Relationship with the clinicOpathological Parameters

**Figure 3:**
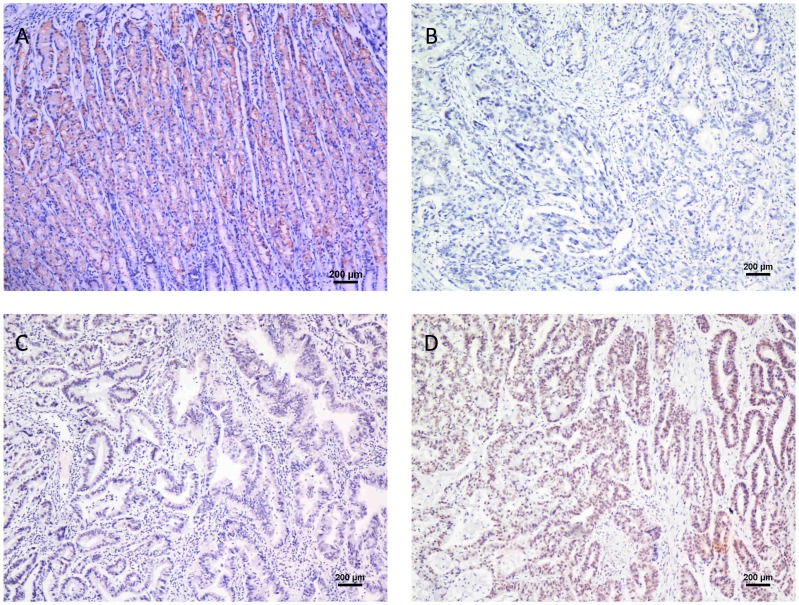
ARID1A protein expression in gastric cancer surgical specimens shown by immunohistochemistry. (A) Strong ARID1A staining was observed in noncancerous gastric mucosa. (B) ARID1A-negative gastric adenocarcinoma, Grade 3. (C) Weak ARID1A staining in gastric adenocarcinoma, Grade 2. (D) Strong ARID1A staining in gastric adenocarcinoma, Grade 1.

To further investigate the clinicopathological and prognostic roles of ARID1A expression, we performed immunohistochemical analyses of the 224 paraffin-embedded gastric cancer tissue blocks. Overall, 115 of 224 (51.3%) cases showed negative ARID1A expression in cancerous tissues (Figure 3B), whereas 109 (48.7%) cases showed positive immunostaining (Figure 3C&D). Normal gastric mucosa showed the strongest ARID1A positive staining (Figure 3A). The correlations between the expression of ARID1A and various clinicopathological parameters are listed in Table 1. The data showed that the loss of ARID1A expression was significantly correlated with depth of tumor infiltration (T stage, *P* = 0.001) and tumor grade (*P* = 0.006), but not with age, gender, tumor size, distant metastasis (M stage), and tumor locus or local lymph node metastasis (N stage).

**Table 1:** Correlation between ARID1A expression and clinicopathological variables of 224 gastric cancer cases.

Clinicopathological parameters	*n* a	ARID1A expression		χ^2^	*P* value
		Positive	Negative		
**All**	224	109	115		
**Age (years)**					
<55	104	46	58	1.525	0.230
≥55	120	63	57		
**Gender**				3.395	0.068
Male	149	66	83		
Female	75	43	32		
**Tumor size**				3.850	0.053
<3 cm	38	24	14		
≥3 cm	186	85	101		
**Tumor infiltration**				16.108	0.001*
T1	32	22	10		
T2	27	18	9		
T3	155	68	87		
T4	10	1	9		
**Local lymph node metastasis**				6.733	0.081
N0	90	49	41		
N1	77	39	38		
N2	32	9	23		
N3	25	12	13		
**Distant metastasis**				3.061	0.102
M0	204	103	101		
M1	20	6	14		
**Grade**				9.812	0.006*
1	7	5	2		
2	41	28	13		
3	176	76	100		

a Numbers of cases in each group. * Statistically significant (*P*<0.05).

### Expression of ARID1A and Clinical Outcome

**Figure 4:**
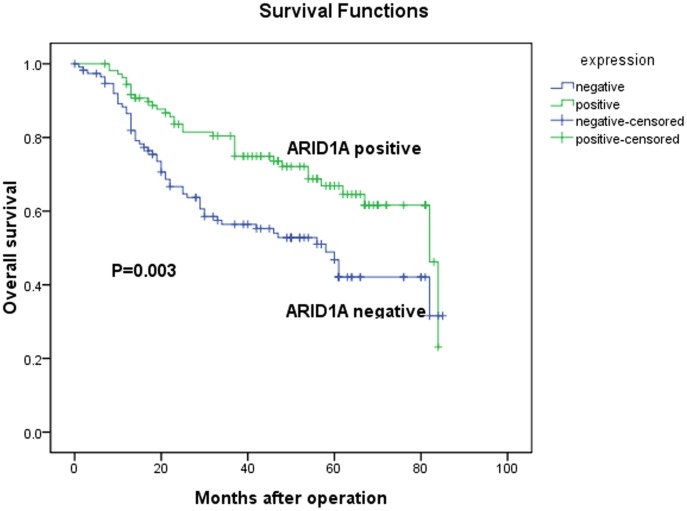
Kaplan-Meier survival curves of gastric cancer patients (n = 224) after gastrectomy. The survival rate of patients in the ARID1A-negative group was significantly lower than that of patients in the ARID1A-positive group (log-rank test, *P* = 0.003).

The 5-year overall survival rates in patients with positive and negative ARID1A expression were 68.8% and 52.2%, respectively. The overall survival of patients with negative ARID1A expression was significantly worse than that of ARID1A-positive patients (*P* = 0.003, log-rank test, Figure 4). Univariate Cox regression analyses showed that depth of tumor infiltration, local lymph node metastasis, distant metastasis, tumor size and ARID1A expression were significantly associated with overall survival (Table 2). Furthermore, a multivariate Cox regression analysis confirmed the depth of tumor infiltration, local lymph node metastasis, distant metastasis and ARID1A expression as independent predictors of the overall survival of gastric cancer patients (Table 2).

**Table 2:** Univariate and multivariate analyses of overall survival of gastric cancer patients.

Variables	*n* a	Univariate analyses			Multivariate analyses		
		HR	(95% CI)	*P* value	HR	(95% CI)	*P* value
**Age (years)**				0.141			
<55	104	1.000					
≥55	120	1.357	0.904–2.309				
**Gender**				0.923			
Female	75	1.000					
Male	149	0.980	0.648–1.482				
**Tumor size**				0.002*			0.479
<3 cm	38	1.000			1.000		
≥3 cm	186	4.218	1.714–10.381		1.450	0.519–4.056	
Tumor infiltration				0.004*			0.044*
T1	32	1.000			1.000		
T2	27	3.056E4	0.000–3.442E66		8.086E3	0.000–1.126E49	
T3	155	9.661E4	0.000–1.086E67		1.897E4	0.000–2.635E49	
T4	10	2.099E5	0.000–2.362E67		4.309E4	0.000–5.999E49	
Local lymph node metastasis				<0.001*			<0.001*
N0	90	1.000			1.000		
N1	77	3.286	1.805–5.984		1.956	1.023–3.743	
N2	32	5.688	3.004–10.768		2.077	1.010–4.271	
N3	25	7.717	3.895–15.288		5.225	2.524–10.818	
Distant metastasis				<0.001*			<0.001*
M0	204	1.000			1.000		
M1	20	6.347	3.854–10.453		5.230	2.998–9.124	
Grade				0.504			
1	7	1.000					
2	41	1.968E4	0.000–1.550E60				
3	176	2.704E4	0.000–2.127E60				
ARID1A				0.003*			0.029*
Negative	115	1.000			1.000		
Positive	109	0.525	0.342–0.805		0.611	0.393–0.950	

HR, hazard ratio; CI, confidence interval;

a Numbers of cases in each group;

* Statistically ignificant (*P*<0.05).

### The Role of *ARID1A* in Cell Proliferation and Colony Formation in MGC803 and GES1 Cell Lines

**Figure 5:**
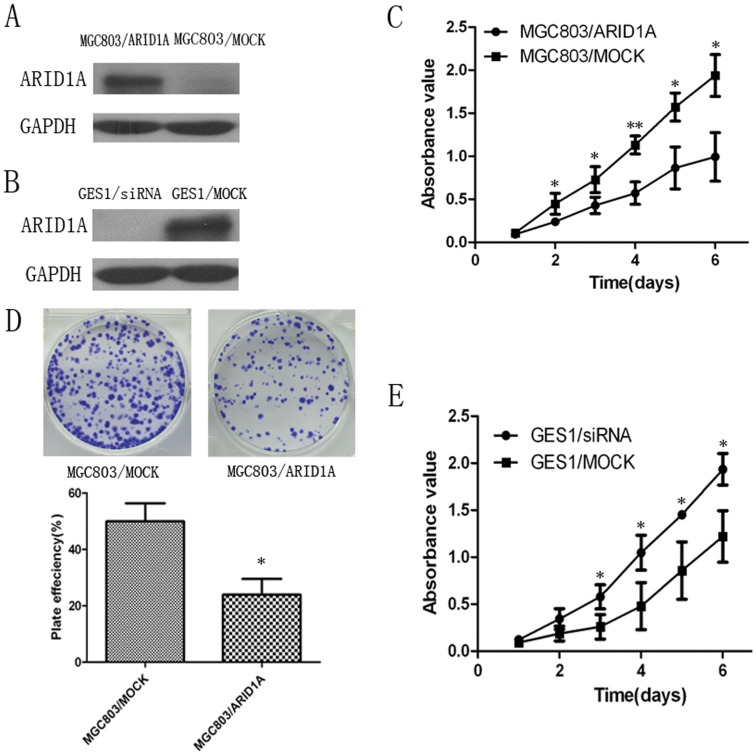
The growth suppressor role of *ARID1A* in cell proliferation and colony formation assays of MGC803 and GES1 cell lines. (A) Western blotting analysis of restoring *ARID1A* expression in MGC803 cells. (B) Western blotting analysis of silenced *ARID1A* expression in GES1 cells. (C) Cell proliferation assay showing the suppressive effect of restoring *ARID1A* expression on the in vitro proliferation of MGC803 cell line. (D) *ARID1A* inhibited colony formation of MGC803 cells. Images are shown on the left and on the right, quantitative analyses of plaque numbers are shown as mean ± SD. (E) Cell proliferation assay showing significantly enhanced proliferation rate of *ARID1A*-silenced GES1 cells compared with mock siRNA treatment GES1 cells. *, *P*<0.05 versus the mock-control; **, *P*<0.01 versus the mock-control.

To evaluate the effects of *ARID1A* on cell proliferation**,** the *ARID1A* expression vector and the control vector were respectively transfected into MGC803 cells. *ARID1A* expression in transfected cells were detected by western blotting (Figure 5A). The cell growth assay revealed that cell growth rate in *ARID1A*-transfected gastric cancer cells were significantly lower than control vector-transfected gastric cancer cells (Figure 5C). Meanwhile, the efficiency of colony formation was significantly (*P* = 0.0379) inhibited in *ARID1A*-transfected gastric cancer cells compared with control vector-transfected gastric cancer cells (Figure 5D). To further confirm the proliferation suppression function of *ARID1A*, we silenced the *ARID1A* expression in GES1 cell line with siRNA. The *ARID1A* expression in transfected cells were detected by western blotting (Figure 5B). We found that silencing the expression of *ARID1A* in GES1 significantly enhanced cell proliferation compared with mock siRNA treatment (Figure 5E).

## Discussion

In spite of great advances in diagnosis and therapy, gastric cancer remains one of the most deadly neoplasms, with a dismal prognosis after radical gastrectomy [14], [15]. The clinical outcome of gastric cancer is determined by a series of tumor characteristics, such as locoregional tumor growth and invasion, differentiation grade, angiogenesis, distant metastasis and cell cycle progression, which are regulated by a variety of related genes, including oncogenes and tumor suppressor genes. Therefore, identification of gastric cancer-specific biomarkers involved in these procedures is very important for diagnosis, therapy and prognosis prediction in clinic.

*ARID1A*, a newly identified tumor suppressor gene which encodes a member of the SWI/SNF complex, has a high mutation frequency in bladder cancer, uterine endometrioid carcinoma, ovarian endometrioid and clear cell carcinoma [11]–[13], [16], [17]. In ovarian clear cell carcinoma, it is reported that *ARID1A* mutation is significantly associated with ARID1A immunoreactivity [18]. Recently, exome sequencing study revealed that *ARID1A* is also frequently mutated in gastric cancer [19], [23]. However, thus far the expression, clinical significance and biological functions of *ARID1A* in gastric cancer have not been explored. Therefore, we evaluated the expression of *ARID1A* in gastric cancer by real-time PCR, western blotting and immunohistochemistry, in addition to its clinicopathological and prognostic significance in a large human sample. Furthermore, using in vitro cell model, we also investigated the tumor suppressor role of *ARID1A* in gastric cells in detail.

In the current study, we demonstrated that *ARID1A* was expressed at both lower mRNA and protein level in gastric cancer tissues than corresponding non-cancerous mucosa. In agreement with these molecular biological findings, immunohisto- chemistry with a anti-ARID1A antibody showed that *ARID1A* was completely silenced in 115 out of 224 patient gastric cancer samples, with positive expression in another 109 patients. Our observation is in agreement with a series of studies revealing that *ARID1A* expression is frequently lost or reduced in a number of cancer tissues and cell lines, such as breast cancer, uterine endometrioid carcinoma, ovarian clear cell and endometrioid carcinoma [13,18,20, and 21].

To date, the causes of *ARID1A* silencing have not been fully elucidated. The existing studies focus on mutations in *ARID1A*, particularly in gynecologic cancers. It is reported that a nonsense or indel mutation of *ARID1A* was correlated with loss or reduction of protein expression in uterine endometrioid carcinoma, ovarian endometrioid carcinoma and clear cell carcinoma [11]–[13], [18]. In an integrated genomic investigation, Mamo *et al*. only found one truncated mutation of *ARID1A* in the T47D breast cancer cell line, without any mutation in the 11 breast cancer samples which showed DNA copy number loss at the 1p36.11 locus adjacent to *ARID1A* [22]. Eight of nine samples with DNA copy number loss at 1p36.11 also have low ARID1A protein expression, suggesting a concordance between DNA copy number loss and *ARID1A* inactivation. In the exome sequencing study by Wang *et al*., a total of 46 mutations was found in 32 out of 109 (29%) gastric cancer samples, with 39 (85%) truncated mutations [19]. Twenty-four (75%) of the 32 gastric cancer samples with *ARID1A* mutations show either loss of or substantially reduced protein expression compared to those without *ARID1A* mutation. In contrast, there are only 6 gastric cancer samples showing absent or weak protein expression in the absence of detectable *ARID1A* mutation, which suggests that other mechanisms may contribute to *ARID1A* inactivation. Recently, another exome sequencing research by Zang *et al*. also showed *ARID1A* mutations in 8% of gastric samples, of which 75% lost or reduced the protein expression [23]. More interestingly, both studies demonstrated higher *ARID1A* alterations in gastric cancer samples with microsatellite instability (MSI) than those with microsatellite stability (MSS). Moreover, the mutation spectrum of *ARID1A* is distinct between the two genetic types of gastric cancer, with most indels in the MSI type and more single-neucliotide variations in the MSS type. MSI is defined as indel mutations within nucleotide repeats (known as microsatellite regions) resulted from DNA mismatch repair gene inactivation-induced replication errors [24]. Proposed as the initiating genomic events of gastric cancer, MSI often leads to accumulation of additional cancer-related genetic instabilities, such as allelic losses and frameshift mutations in genes involved in cell proliferation regulation, apoptosis and DNA repair. It has been reported that MSI occurs in 25% to 50% of sporadic gastric cancer, defining a unique genetic type disease with different clinicopathological features [24]. In the study of Wang *et al*., the indel mutation rate (78%) of *ARID1A* in MSI gastric cancer is comparable to that of *TGFBR2* in MSI colon cancer, a well-established and functionally validated driver gene inactivated by MSI [25]. These data indicate that the mutation of *ARID1A* together with MSI may play an important role in gastric carcinogenesis. Therefore, the relationship between *ARID1A* alterations and MSI status in gastric cancer, as well as its clinicopathological significance, needs further investigation in the future research.

In the study by Wang *et al.*, the expression of *ARID1A* was only detected in a small-size sample (32 cases), and there was no further exploration of its clinical significance [19]. Here, in a larger gastric cancer population (224 cases), we found that the loss of *ARID1A* expression was significantly correlated with a higher T stage of gastric cancer, implying that absence of *ARID1A* expression may promote tumor growth and invasion. In addition, we detected lower ARID1A immunoreactivity in poorly differentiated gastric cancer tissues than in well-differentiated ones, suggesting that decreased *ARID1A* expression might play a role in tumor de-differentiation. Consistent with our findings, other investigators also found that decreased *ARID1A* expression is significantly associated with a higher grade of breast cancer [22], as well as a higher FIGO stage in ovarian clear cell carcinoma [21]. ARID1A promotes the formation of BRG1 or BRM-contained SWI/SNF chromatin remodeling complexes, which are essential for normal cell cycle arrest [9], [26].

A Kaplan-Meier survival analysis showed a significant correlation between the loss of *ARID1A* expression and poorer clinical outcome of gastric cancer patients after radical operation. Cox hazard ratio regression analyses further demonstrated that the *ARID1A* expression level was an independent risk factor for survival, suggesting that it may serve as a valuable prognostic biomarker for gastric cancer patients after surgery and a potential target for gene therapy in the treatment of gastric cancer. In ovarian clear cell carcinoma, it was also reported that patients with positive *ARID1A* expression had a longer progression-free survival than those with negative *ARID1A* expression [21]. Moreover, loss of *ARID1A* expression is significantly correlated with chemoresistance in ovarian clear cell carcinoma, which is also associated with a poor prognosis of cancer. These data suggest that *ARID1A* expression and mutation examination might be helpful to guiding clinical management. Taken together, our observations that the loss of *ARID1A* expression in gastric cancer is associated with more malignant phenotypes and a worse prognosis imply that it may play a tumor suppressor role in gastric carcinogenesis.

We further investigated the functional role of *ARID1A* in gastric cell lines. Restoring *ARID1A* expression in gastric cancer cells significantly inhibited cell proliferation and colony formation. Silencing the expression of *ARID1A* in gastric epithelial cells significantly enhanced the cell growth rate. These results indicated that *ARID1A* may play an import role in inhibiting tumor cell growth. Recently, functional assays of *ARID1A* in gastric cancer cell lines by Zang *et al*. suggested that *ARID1A* exert tumor-suppressor activity [23]. Guan *et al*. demonstrated that restoring the expression of wild-type *ARID1A* is sufficient to suppress the proliferation and tumorigenecity of xenografts with human ovarian cancer cell lines harboring *ARID1A* mutations, while RNA interference-mediated *ARID1A* silencing enhances cellular proliferation and tumorigenicity in two non-transformed human ovarian epithelial cell lines, IOSE-80PC and OSE4 [27]. These data, together with ours, suggest that loss of *ARID1A* may play an important role in the process of carcinogenesis.

In conclusion, we have demonstrated the loss of *ARID1A* expression in gastric cancer and its correlation with a more malignant phenotype and poorer prognosis in a large number of clinical samples. In addition, we proved that *ARID1A* can inhibit tumor cell growth and colony formation in vitro. To the best of our knowledge, the data generated in the current study represent the first report correlating the presence of *ARID1A* with clinicopathological characteristics and the overall survival of gastric cancer patients. Taken together with the results of Wang *et al.* and Zang *et al*. [19], [23], we further confirmed that *ARID1A* might serve as a candidate tumor suppressor and prognostic biomarker in gastric carcinogenesis.

## Materials and Methods

### Ethics Statement

The research was approved by the Ethics Committee of Sun Yat-sen University Cancer Center, and written informed consent was obtained from each patient involved in the study.

### Cell Lines and Culture Conditions

The gastric cancer cell lines, SGC7901, AGS, MGC803, and the gastric epithelial mucosa cell line GES1 were obtained from the Committee of Type Culture Collection of Chinese Academy of Sciences (Shanghai, China). The cell lines were cultured in RPMI 1640 media supplied with 10% heat-inactive fetal bovine serum (FBS). The cells were incubated at 37°C in a humidified chamber containing 5% CO_2_.

### Human Tissue Sample*s*

A total of 66 paired cancerous and matched adjacent noncancerous gastric mucosa tissues were collected from gastric cancer patients undergoing gastrectomy at Sun Yat-sen University Cancer Center between 2009 and 2011, and the diagnosis was confirmed by pathological examination. The 25 paired cancerous and corresponding adjacent noncancerous gastric mucosa tissues used to detect the ARID1A protein expression in western blotting were selected from the 66 paired samples. After surgical resection, fresh tissues were immediately immerged in RNAlater (Ambion, Inc., USA) to avoid RNA degradation, stored at 4°C overnight to allow thorough penetration of RNAlater into the tissue and then frozen at −80°C until RNA and protein extraction was performed. Another 224 paraffin-embedded primary gastric carcinoma samples which had been collected between 2003 and 2005, were obtained from the Sun Yat-sen University Cancer Center. None of these patients had received radiotherapy or chemotherapy prior to surgery. The follow-up data of the gastric cancer patients in this study are available and complete. Postoperative follow-up occurred at our outpatient department and included clinical and laboratory examinations every 3 months for the first 2 years, every 6 months during the third to fifth years, annually for an additional 5 years or until patient death, whichever occurred first. The histopathological type and stage of gastric cancer were determined according to the criteria of the World Health Organization classification and the TNM stage set out by the Union for International Cancer Control.

### Extraction of Total RNA and Real-time Quantitative PCR

Total RNA was extracted using TRIzol (Invitrogen, Carlsbad, California, USA) according to the manufacturer’s protocol. Total RNA concentration was assessed by measuring absorbance at 260 nm using a NANO DROP spectrophotometer (ND-1000, Thermo Scientific, USA). Reverse transcription (RT) to synthesize the first-strand of cDNA was performed with 2 µg of total RNA treated with M-MLV reverse transcriptase (Promega, USA) according to the manufacturer’s recommendations. The resulting cDNA was then subjected to real-time quantitative PCR for evaluation of the relative mRNA levels of *ARID1A* and *GAPDH* (glyceraldehyde-3-phosphate dehydrogenase, as an internal control) with the following primers: *ARID1A* forward: 5′-CTTCAACCTCAGTCAGCTCCCA-3′, and reverse: 5′-GGTCACCCACCTCATACTCCTTT-3′; *GAPDH* forward: 5′-CTCCTCCTGTTCGACAGTCAGC-3′, and reverse: 5′-CCCAATACGACCAAATCCGTT-3′. Gene-specific amplification was performed using an ABI 7900HT real-time PCR system (Life Technologies, Carlsbad, California, USA) with a 15 µl PCR mix containing 0.5 µl of cDNA, 7.5 µl of 2 x SYBR Green master mix (Invitrogen, Carlsbad, California, USA), and 200 nM of the appropriate oligonucleotide primers. The mix was preheated at 95°C (10 min) and then amplified at 95°C (30 sec) and 60°C (1 min) for 45 cycles. The resolution curve was measured at 95°C for 15 sec, 60°C for 15 sec and 95°C for 15 sec. The Ct (threshold cycle) value of each sample was calculated from the threshold cycles with the instrument’s software (SDS 2.3), and the relative expression of *ARID1A* mRNA was normalized to the *GAPDH* value. Data were analyzed using the comparative threshold cycle (2^-ΔCT^) method.

### Western Blotting Analysis

The homogenized gastric cancer samples, including tumor and nontumor tissues, as well as cell lines, were lysed in RIPA lysis buffer, and the lysates were harvested by centrifugation (12,000 rpm) at 4°C for 30 min. Approximately 50 µg protein samples were then separated by electrophoresis in a 12% sodium dodecyl sulfate polyacrylamide gel and transferred onto a polyvinylidene fluoride membrane. After blocking the non-specific binding sites for 60 min with 5% non-fat milk, the membranes were incubated overnight at 4°C with a mouse monoclonal antibody against ARID1A (Abgent Primary Antibody Company, USA, at a 1∶1000 dilution). The membranes were then washed three times with TBST (tris-buffered saline with tween-20) for 10 min and probed with the horseradish peroxidase (HRP)-conjugated rabbit anti-mouse IgG antibody (Immunology Consultants Laboratory, USA, at a 1∶2000 dilution) at 37°C for 1 hour. After three washes, the membranes were developed by an enhanced chemiluminescence system (Cell Signaling Technology, Danvers, Massachusetts, USA). The band intensity was measured by densitometry using the Quantity One software (Bio-Rad Laboratories, Inc. Hercules, CA, USA). The protein levels were normalized to that of GAPDH detected using mouse anti-human GAPDH monoclonal antibody (Shanghai Kangchen, China, at a 1∶10000 dilution).

### Immunohistochemistry Analysis

The tissue sections were deparaffinized with dimethylbenzene and rehydrated through 100%, 95%, 90%, 80% and 70% ethanol. After three washes in PBS (phosphate-buffered saline), the slides were boiled in antigen retrieval buffer containing 0.01 M sodium citrate-hydrochloric acid (pH = 6.0) for 15 min in a microwave oven. After rinsing with PBS, the tissue sections were incubated with primary antibody and the slides were then rinsed in 3% peroxidase quenching solution (Invitrogen) to block endogenous peroxidase. The sections were then incubated with a mouse monoclonal antibody against ARID1A (Abgent Primary Antibody Company, USA, at a 1∶300 dilution) at 4°C overnight and then incubated with horseradish peroxidase (HRP) (ChemMateTM DAKO EnVisionTM Detection Kit) at room temperature for 30 min. After washing in PBS, the visualization signal was developed with 3, 3′-diaminobenzidine (DAB) solution, and all of the slides were counterstained with hematoxylin. As negative controls, adjacent sections were processed as described above except that they were incubated overnight at 4°C in blocking solution without the primary antibody.

The total ARID1A immunostaining score was calculated as the sum of the percent of positively stained tumor cells and the staining intensity. Briefly, the percentage of positive staining was scored as 0 (0–9%, negative), 1 (10%–25%, sporadic), 2 (26%–50%, focal) or 3 (51%–100%, diffuse), and the intensity as 0 (no staining), 1 (weak staining), 2 (moderate staining) and 3 (dark staining). The total immunostaining score was calculated with the value of percent positivity score × staining intensity score, which ranged from 0 to 9. The expression level of ARID1A was defined as following: “−” (negative, score 0), “+” (weakly positive, score 1–3), “++” (positive, score 4–6), “+++” (strongly positive, score 7–9). Based on the ARID1A expression levels, the gastric cancer patients were divided into two groups: negative ARID1A expression group (ARID1A-) and positive ARID1A expression group (ARID1A+, ARID1A++ or ARID1A+++).

### Expression Plasmid and Transient Transfections

A eukaryotic expression plasmid pCMV6-Entry containing the full-length of human *ARID1A* cDNA was obtained from the Asbio Technology Company (Guangzhou, China). Empty vector was used as negative control. MGC803 cells were cultured in 6-well plates until they reached 85–90% confluence, and then transient transfections were performed using Lipofectamine 2000 (Invitrogen) according to the manufacturer’s instructions. Forty-eight hours after transfection, gene expression was examined by western blotting analysis. And then, cell proliferation and colony formation were performed.

### RNA Oligonucleotides and Cell Transfections

For knockdown of *ARID1A* expression, the siRNAs were synthesized by GenePharma Company (Shanghai, China). The siRNA sequences were as follows: siRNA-*ARID1A*, sense: 5′GCCCUAACAUGGCCAAUAUTT3′, antisense: 5′AUAUUGGCCAUGUUAGGGCTT3′. The negative control (NC), sense: 5′UUCUCCGAACGUGUCACGUTT3′, antisense: 5′ACGUGACACGUUCGGAGAATT3′. 400 pmol siRNA-*ARID1A* or NC were transfected into 2×10^5^ GES1 cells using Lipofectamine RNAi MAX reagent (Invitrogen, USA) according to the manufacturer’s protocol. After that, cell proliferation was then performed.

### Proliferation Assay

Cell growth rate of MGC803 or GES1 cells was detected by MTS cell proliferation assay. Cells were seeded in a 96-well plate at a density of 5×10^2^ cells per well. The cell growth rate was detected using cell proliferation MTS kit according to the manufacturer's instruction (Promega, USA). Each experiment was performed in triplicate.

### Colony Formation Assay

For the colony formation assay, *ARID1A*-expressing MGC803 cells or control MGC803 cells were plated in a 6-well plate at a density of 5×10^2^ cells per well. After 10 days of culture, surviving colonies (>50 cells per colony) were counted with crystal violet (0.5%) staining. Colony-forming efficiency (CFE %) was defined as the ratio of the number of colonies formed in culture to the number of cells inoculated. The experiment was done in triplicate.

### Statistical Analysis

Differences in mRNA and protein expression between tumor samples and the paired adjacent non-tumor tissue samples were evaluated with the paired-samples t-test. The χ^2^ test was used to analyze the relationships between ARID1A expression and various clinicopathological parameters. Survival curves were calculated using the Kaplan–Meier method and compared by the log-rank test. The Cox proportional hazard regression model was used for univariate and multivariate analyses to study the effects of the clinicopathological variables and ARID1A expression on survival. The two-tailed unpaired Student’s *t* test was used to assess differences in cell growth rate and colony formation. Statistical analyses were performed with the Statistical Package for the Social Sciences, version 17.0 (SPSS Inc., Chicago, IL, USA), and a two-sided *P* value less than 0.05 was considered to be statistically significant.
